# Polidocanol inhibits *Enterococcus faecalis* virulence factors by targeting *fsr* quorum sensing system

**DOI:** 10.1186/s12866-024-03548-2

**Published:** 2024-10-16

**Authors:** Dina Ashraf, Mona I. Shaaban, Ramadan Hassan, Abeer M. Abd El-Aziz

**Affiliations:** Department of Microbiology and Immunology, Faculty of Pharmacy, Mansoura University, Mansoura, 35516 Egypt

**Keywords:** *Enterococcus faecalis*, Quorum sensing, Gelatinase, Virulence

## Abstract

**Background:**

The wide spread of antimicrobial resistance in *Enterococcus faecalis* is a critical global concern, leading to increasingly limited treatment options. The *fsr* quorum sensing (QS) plays a critical role in the pathogenicity of *E. faecalis*, allowing bacteria to coordinate gene expression and regulate many virulence factors. Therefore, *fsr* QS of *E. faecalis* represents a potential therapeutic target that provides an effective strategy to treat antibiotic-resistant infections induced by *E. faecalis*.

**Methods:**

In this study, distribution of different virulence factors including, gelatinase, protease, cell surface hydrophobicity and biofilm formation in sixty clinical isolates of *Enterococcus faecalis* was investigated. Sixty-six compounds were tested for their activity against *fsr* QS. The minimal inhibitory concentration of the tested compounds was evaluated using the microbroth dilution method. The effect of sub-inhibitory concentrations of the tested compounds on *fsr* QS was investigated using the gelatinase assay method. Additionally, the effect of potential QS inhibitor on the virulence factors was estimated. Quantitative real-time PCR was used to investigate the effect of the potential inhibitor on *fsr* QS related genes (*fsrB-fsrC*) and (*gelE-sprE*) and virulence associated genes including, *asa1* and *epbA*.

**Results:**

The assessment of polidocanol activity against the *fsr* QS system was demonstrated by studying its effect on gelatinase production in *E. faecalis* clinical isolates. Sub-lethal concentrations of polidocanol showed a significant reduction in gelatinase and protease production by 54% to 70% and 64% to 85%, respectively. Additionally, it significantly reduced biofilm formation (*P* < 0.01) and interrupted mature biofilm at concentrations of ½, 1 × and 2 × MIC. Furthermore, polidocanol significantly decreased cell surface hydrophobicity (*P* < 0.01). Polidocanol at ½ MIC showed a significant reduction in the expression of QS genes including *fsrB*, *fsrC*, *gelE* and *sprE* by 57% to 97% without affecting bacterial viability. Moreover, it reduced the expression of virulence associated genes (*asa1* and *epbA*) (*P* < 0.01).

**Conclusion:**

Polidocanol appears to be a promising option for treating of *E. faecalis* infections by targeting the *fsr* QS system and exhibiting anti-biofilm activity.

**Supplementary Information:**

The online version contains supplementary material available at 10.1186/s12866-024-03548-2.

## Background

*Enterococcus faecalis* is a Gram-positive bacterium that typically resides in the human gastrointestinal tract (GIT) as a commensal organism. This bacterium can cause various diseases in humans, including urinary tract infections, joint infections, abdominal-pelvic infections, bacteremia, and endocarditis. These diseases can vary in severity and pose a life-threatening risk, particularly for individuals with compromised immune systems [1]. Furthermore, enterococci have emerged as one of the most frequent causes of nosocomial infections since the 1970s, with *E. faecalis* being responsible for approximately 60% of these infections [2]. The rise of *E. faecalis* infection as leading nosocomial pathogen has paralleled the emergence of strains that are resistant to a wide range of antimicrobial drugs including vancomycin, which is considered the last defense line [3].

*E. faecalis* has developed an array of virulence factors to successfully colonize and survive within the host, evade the immune system, and induce tissue damage. These factors include adherence, biofilm formation, antiphagocytosis, exoenzyme, and exotoxin [4].

The main proteases produced by *E. faecalis* include gelatinase enzyme (GelE) and serine protease (SprE)*.* GelE is classified as metalloprotease II that can break down gelatin, fibrin, fibrinogen, collagen, hemoglobin, complement components, casein, endothelin-1, and other small peptides [5]. Additionally, GelE contributes to the pathogenesis *of E. faecalis* by triggering the autolysin responsible for biofilm formation [6], while SprE is a glutamyl endopeptidase I that hydrolyze casein and contributes to *E. faecalis* pathogenesis in various hosts [7].

The *E. faecalis* system regulator* (fsr*) is the major quorum-sensing (QS) system in *E. faecalis,* comprising four genes: *fsrA*, *fsrB*, *fsrC*, and *fsrD*. The *fsr* positively controls the production of gelatinase and serine protease, encoded by *gelE* and *sprE*, respectively [4]. It is also responsible for controlling *E. faecalis* biofilm formation by regulating gelatinase production [8]. The *fsrA* gene encodes the FsrA protein, a member of the LytTR family of DNA-binding domains. When phosphorylated, FsrA binds to LytTR-binding sites in the upstream regions of *fsrB* and *gelE*, indicating its role as a response regulator in the *fsr* system [9]. FsrB, encoded by the *fsrB* gene, is a transmembrane protein belonging to the accessory gene regulator protein B (AgrB) family, responsible for processing the propeptide FsrD (encoded by *fsrD*) to produce an autoinducer called gelatinase biosynthesis-activating pheromone (GBAP). This cyclic peptide contains 11 amino acid residues which is then exported out of the cell [10]. The *fsrC* gene encodes FsrC, a transmembrane histidine protein kinase that serves as the sensor-transmitter component of the *fsr* operon [11]. Once the extracellular concentration of GBAP reaches a certain threshold level, it activates a two-component regulatory system composed of FsrC and FsrA. FsrC phosphorylates the intracellular FsrA in response to extracellular GBAP. Activated FsrA stimulates the expression of the *fsrBDC* transcript, which is involved in an autoregulatory circuit that amplifies GBAP signaling and leads to the induction of *gelE*-*sprE* transcription [7, 12].

Since enterococci able to resist to antibiotics of all classes that have so far been introduced to practice, there is a growing interest in exploring alternative therapies to conventional antimicrobials as anti-virulent therapy. Interfering with the QS system in order to combat *E. faecalis* infections shows great promise. Nakayama found that a secondary metabolite produced by actinomycetes, siamycin I, has inhibitory effects on FsrC, a component of the *fsr* QS system [12]. Two compounds, Y67-1 and Y67-2, were also extracted from actinomycetes through high throughput screening analysis. Both of these compounds are receptor antagonists of FsrC [13]. A different study with promising results discovered that ambuic acid, a secondary metabolite, produced by fungi, was able to inhibit the proteolytic modification of FsrD by binding to FsrB [14].

In this particular context, the research focused on the assessment of *fsr* related virulence factors among *E. faecalis* clinical isolates including, gelatinase, protease, cell surface hydrophobicity and biofilm formation. Moreover, clinically approved medications were evaluated for their potential inhibitory effect on the *E. faecalis fsr* QS system. The effect of the potential inhibitor on *fsr* related virulence factors was investigated phenotypically and confirmed on the molecular level by qRT-PCR.

## Methods

### Bacterial strains

Sixty clinical specimens, including blood, urine, stool and pus samples, were collected from patients at some healthcare facilities, including Mansoura University Hospitals and Kasr Al-Ainy Hospital. This collection spanned between February 2021 and July 2021. All specimens were collected using a protocol approved by the Research Ethics Committee of Faculty of Pharmacy, Mansoura University with the ethical codes 2023—157. The isolates were phenotypically identified as *Enterococcus* by streaking them on bile esculin agar (Biolab®, Hungary) [15]. They were further genotypically verified as being of the species *faecalis* through PCR analyses of species-specific eda-genes *(eda1* and *eda2)*, which encode the enzymes involved in the Entner–Doudoroff pathway, using primer pairs listed in Table 1.

**Table 1:** PCR primers utilized in PCR and qRT-PCR

Genes	Gene name	Type	Nucleotide sequence (5` to 3`)	Amplicon size (bp)	Temperature	Reference
***E. faecalis* species identification genes**	***eda-1***	***Fw***	GGGGACAGTTTTGGATGCTA	404	51	[16]
		***Rv***	TCCATATAGGCTTGGGCAAC			
	***eda-2***	***Fw***	GCCGAAGCTTCATCTTCTTG	389		
		***Rv***	AGGCGCAGGAACTGTTAGAA			
***fsr* QS related genes**	***fsrB***	***Fw***	TCTTCTGTGAGCTTACCGTTT	210	61	[17]
		***Rv***	GACCGTAGAGTATTACTGAAGCA			
	***fsrC***	***Fw***	TGACGAAACATCGCTAGCTC	194	61	[18]
		***Rv***	ATGCGAGGATTTGTCACGGT			
	***gelE***	***Fw***	AGTGAACGCTACAGATGGAAC	145	60	[12]
		***Rv***	CGTTCCGTGTAAAGCAATTCC			
	***sprE***	***Fw***	AAGATCGTTACTGGACCCTGAG	239	59	[19]
		***Rv***	GACCTGGATAAAACCAAGCATC			
**Virulence determinant genes**	***asa1***	***Fw***	GCACGCTATTACGAACTATGACC	378	57	[20]
		***Rv***	TAAGAAAGAACATCACCACGAGC			
	***ebpA***	***Fw***	CAACAACACCAGGGCTTTTTG	126	62	[21]
		***Rv***	ACCGGACCAGTCAACGACTAAG			
**Housekeeping gene**	***16 sRNA***	***Fw***	GTAGCGGTGAAATGCGTAGATA	160	61	[19]
		***Rv***	GAAACCCTCCAACACTTAGCAC			

*Fw:* Forward

*Rv:* Reverse

*bp:* Base pair

All isolates were routinely grown in double strength tryptic soy broth (TSB) **(**Oxoid™, UK**)** at 37 °C for approximately 24 h with shaking (150 rpm) and kept in 30% (v/v) glycerol stocks at − 80 °C.

## Phenotypic characterization of the tested isolates

### Assay of gelatinase and serine protease production

Gelatinase production among *E. faecalis* isolates was qualitatively investigated using nutrient agar containing 3% (w/v) gelatin. Inoculated nutrient gelatin plates were incubated overnight at 37 °C and were then flooded with a saturated solution of ammonium sulphate. The presence of a distinct clear zone surrounding the colonies indicated gelatinase production [22].

Whereas, protease activity was detected using agar containing 1.5% (w/v) skimmed milk. A transparent zone around colonies, observed after 24 h of incubation at 37 °C, was recorded as a positive indication of protease activity [23].

### Detection of hydrophobicity of *E. faecalis* isolates

The surface hydrophobicity of the bacterial cell was assessed using BATH test (bacterial adhesion to hydrocarbons) according to the method of Rosenberg and coauthors [24]. This assay quantifies the reduction in culture density within the aqueous solution following mixing with and separation of the hydrocarbon layer. Cells with low cell surface hydrophobicity, which are hydrophilic, will remain in the aqueous layer. In contrast, cells with higher cell surface hydrophobicity, which are hydrophobic, will migrate into the hydrocarbon layer, leading to a decrease in culture density in the aqueous layer [24, 25]. Bacterial cells were collected by centrifugation at 3000 rpm at 4 °C for 15 min. Pellets were washed twice with phosphate buffer saline (PBS) (10 mM Na_2_PO_4_, 1.8 mM KH_2_PO_4_, 137 mM NaCl, and 2.7 mM KCl, pH 7.0) and resuspended in PBS to achieve an OD_520 nm_ of 1 (OD_i_). The bacterial suspension was then overlaid with 250 µL of xylene (NATCO. Laboratory Chemicals, India) with shaking for 2 min. The suspension was initially incubated at room temperature for 15 min and then incubated at 37 °C for 30 min. Subsequently, the absorbance (OD_f_) of the aqueous layer was determined at 520 nm. The results were presented as the percentage of cells extracted from the aqueous phase, calculated using the equation: % adherence = [(1 -OD_f_ /OD_i_)] × 100. The bacterial cell surface hydrophobicity was categorized as strongly hydrophobic (> 50%), moderately hydrophobic (20—50%) and hydrophilic (< 20%) [25].

### Biofilm assay

The capacity of the *E. faecalis* isolates to produce biofilm was evaluated using biofilm plate assay [26]. Briefly, the isolates were grown overnight in TSB at 37 °C. Then, the cultures were adjusted to 10^8^ CFU/mL and 20 µL was inoculated into 180 µL of fresh TSB per well. Following a 24 h incubation at 37 °C, the culture medium was discarded and the plates were rinsed three times with PBS to remove non-adherent bacterial cells. Meanwhile, the cells that adhere to the surface were treated with absolute methanol for 15 min for fixation. Each well was then stained by 2% (w/v) crystal violet for 20 min. Subsequently, the plates were washed with distilled water thrice and were air dried. The bound dye was resolubilized by adding 33% (v/v) glacial acetic acid per well and the optical density was measured at 490 nm using a microtiter plate reader (Bio Tek instruments E1800, 29,274, USA). All measurements were conducted in quadruplicates and the mean values of all measurements were reported. The results were interpreted in accordance with Emilia [27]. The cut-off OD (OD_C_) was determined by adding three times the standard deviation to the average OD of the negative control. The average of the OD values for each isolate was subtracted from the average of the OD of the negative control (ODi), and then compared to the value of OD_C_. All *E. faecalis* isolates were classified into four categories: non-adherent (ODi ≤ OD_C_), weakly adherent (OD_C_ < ODi ≤ 2OD_C_), moderately adherent (2OD_C_ < ODi ≤ 4OD_C_), or strongly adherent (4OD_C_ < ODi).

## Molecular detection of *fsr* QS and virulence factors genes

Four isolated *E. faecalis* isolates (Ef10, Ef33, Ef35, and Ef38) possessing *eda1* and *eda2* species specific genes characterized by their ability to produce gelatinase and protease enzymes were selected. The presence of *fsr* QS related genes (*fsrB* and *fsrC*), gelatinase (*gelE*), serine protease (*sprE*) and some virulence factors related genes including the pilus protein gene (*ebpA*) and the aggregation substance gene (*asa1*) was identified through PCR using the oligonucleotide primers listed in Table 1. Bacterial DNA was prepared by boiling fresh bacterial colonies suspended in RNase and DNase free water for 10 min [28]. PCR amplification was performed using a ProFlexTM PCR System (Thermo Fisher Scientific, USA). The amplification protocol consisted of an initial denaturation at 95 °C for 2 min, followed by 40 cycles of denaturation at 95 °C for 30 s, annealing at temperatures specified in Table 1 for each primer pair for 30 s, extension at 72 °C for 30 s, and a final extension step at 72 °C for 5 min. The PCR products were subsequently examined using agarose gel electrophoresis and observed under UV light.

## Screening of different compounds for targeting *E. faecalis fsr* QS system

In this study, sixty-six compounds (Supplementary Table 1), were screened for their *fsr* QS inhibition activity against two clinically isolated *E. faecalis* strains (Ef33 and Ef35). All experiments were conducted three times using separate tests and each sample was tested in triplicate.

### Detection of minimum inhibitory concentration of the tested compounds

The MIC assay was conducted using the broth micro-dilution method outlined by the Clinical and Laboratory Standards Institute [29] to assess the antibacterial activity of the sixty-six tested compounds against *E. faecalis* strains (Ef33 and Ef35). In a 96-well micro-test plate, a series of two-fold serial dilutions for each compound were prepared in Mueller Hinton broth (MHB) (Oxoid™, UK). A 10 µL inoculum of the culture, containing 5 × 10^6^ CFU/mL, was added to each dilution. The micro-plates were then incubated at 37 °C for 24 h. MIC values were measured using a spectrophotometer at OD_600 nm_ to determine the lowest concentration that effectively hindered the bacterial growth [30].

### Gelatinase inhibition assay

The impact of sublethal concentrations (½ and ¼ MIC) of each tested compound on gelatinase production was measured using a semi-quantitative gelatinase assay method [31]. Three microliters of grown overnight culture with (OD_600 nm_ = 0.01) were inoculated into 500 µL of TSB medium with sub-lethal concentrations of each compound and untreated cultures were prepared by inoculating TSB without treatment [12]. After 5 h of incubation at 37 ºC, the cell free supernatants from both treated and untreated cultures were applied to cups made in gelatin agar plates. The plates were incubated at 37 °C for 24 h and the zone of clearance was measured by precipitating unhydrolyzed gelatin using saturated ammonium sulfate solution.

## Growth inhibition assay of polidocanol

A comparative analysis was conducted to assess the growth inhibitory effect of sub-inhibitory concentration of polidocanol (½ MIC) (Docavarico, AMOUN, Egypt) on *E. faecalis* isolates (Ef33 and Ef35). The viable colonies treated with this concentration were enumerated and compared to the count of untreated *E. faecalis* (Ef33 and Ef35), employing the surface drop method [32]. TSB with polidocanol (½ MIC) and a control without polidocanol were inoculated with an overnight culture of *E. faecalis*. The cultures were then incubated at 37 °C for 24 h. Additionally, the growth rate of both untreated *E. faecalis* and cells treated with ½ MIC of polidocanol was observed. Samples were collected at different time intervals and the OD_600 nm_ was measured for both treated and untreated cultures [33, 34].

## The impact of polidocanol as potential inhibitor on *E. faecalis* virulence factors

To study the effect of polidocanol on *E. faecalis* virulence factors, overnight culture of the selected four *E. faecalis* isolates (Ef10, Ef33, Ef35 and Ef38) were inoculated into TSB medium containing sub-lethal concentrations of polidocanol (½ and ¼ MIC). For comparison, untreated cultures were prepared by inoculating TSB without any drugs.

### Gelatinase production assay

To confirm the effect of polidocanol on gelatinase activity, a quantitative assay was performed using azocoll (Azocoll™ Substrate, < 50 Mesh, Sigma-Aldrich, USA) as a substrate [11]. Azocoll (0.25 g) was suspended in 50 mL of 50 mM Tris–HCl buffer (pH 7.8) containing 1 mM CaCl_2_ and incubated statically at 37 °C for 90 min. Then the supernatant was decanted and the precipitate was resuspended in the same buffer. For the assay, 0.5 mL of this azocoll suspension was transferred to an Eppendorf tube and incubated at 37 °C for 15 min with shaking at 170 rpm. To investigate the inhibition of gelatinase production, 25 μL of both treated and untreated *E. faecalis* culture supernatants were added to the preincubated azocoll suspension. The mixture was further incubated for 4 h with shaking at 170 rpm, then centrifuged at 9000 rpm for 5 min, and the OD_520 nm_ of the supernatant was subsequently measured.

### Quantitative assay of proteolytic activity

The impact of sub-MIC of polidocanol on protease activity was quantitatively assayed using skimmed milk assay method [35]. The cell free supernatants of both treated and untreated *E. faecalis* cultures (1 mL) were incubated with 1.5% skimmed milk solution (1 mL) at 37 °C for 2 h. The OD of the supernatant was then measured at 600 nm to determine the degree of proteolytic activity, which was detected by assessment of the digestion of milk protein casein in the reaction mixture.

### Bacterial cell surface hydrophobicity assay

The effect of polidocanol on hydrophobicity of cell surface was assessed by measuring the affinity of *E. faecalis* isolates no. Ef10, Ef33, Ef35 and Ef38 towards xylene after exposure to sub-lethal concentrations (½ and ¼ MIC) of polidocanol as previously described. The percentages of cells attached to xylene in treated and untreated samples were compared [36].

### The impact of polidocanol on biofilm formation

In this research, the impact of polidocanol on the formation of *E. faecalis* biofilm was assessed. The polystyrene plates were inoculated with overnight bacterial suspensions, which were adjusted to 10^8^ CFU/mL in fresh TSB for the control groups and in TSB with sub-lethal concentrations (½ and ¼ MIC) of polidocanol for the treated groups. They were then incubated for 24 h at 37 ºC. After incubation, the biofilms were stained using the previously described crystal violet assay [26].

To assess the impact of polidocanol on mature biofilm, different concentrations of polidocanol (¼, ½, 1 × and 2 × MIC) were applied to bacterial biofilms generated in microtiter plates. Untreated wells filled with 200 µL PBS served as the control. After incubation at 37 °C for 24 h, the wells were drained and rinsed with PBS. The remaining adherent cells were fixed with absolute methanol, stained with crystal violet, and resolubilized with glacial acetic acid. The optical density was then measured at 490 nm [37, 38].

The Tetrazolium chloride (TTC) reduction assay, using (2,3,5-Triphenyltetrazolium, Technical, Fisher Chemical™, USA), was utilized to investigate the effect of polidocanol on the metabolic activity of cells in *E. faecalis* biofilms [39, 40]. The level of biofilm viability was assessed colorimetrically by quantifying the production of reduced 1,3,5-triphenylformazan, which is generated by metabolically active biofilm cells and appears red. Biofilms were formed in flat-bottomed microtiter plates using untreated and polidocanol treated bacterial cells. After removing the planktonic cells and washing the wells twice with PBS, 250 µL of a 0.01% TTC solution was added to each well. The plates were then incubated in the dark at 37 °C for 24 h. Following incubation, the TTC solution was removed, and the wells were allowed to air dry. To dissolve the metabolized TTC dye, 150 µL of an 80:20 solution of ethanol and acetone was added to each well, and the optical OD of the solution was measured at 600 nm.

## Gene expression analysis by quantitative real-time polymerase chain reaction (qrt-PCR)

The impact of polidocanol on the expression of certain QS and virulence genes was assessed using qRT-PCR. *E. faecalis* isolates Ef33 and Ef35 were exposed to polidocanol (½ MIC). Total RNA was extracted from both treated and untreated isolates using TRIzol (Sigma Chemicals, USA. *E. faecalis* isolates Ef33 and Ef35 were grown in TSB medium with and without ½ MIC of polidocanol for 10 h until they became in the exponential phase (OD_600 nm_ = 0.5–0.6). The bacterial cells were then collected by centrifugation at 10,000 rpm for 20 min and then resuspended in TRIzol reagent. The purified RNA was dissolved in diethyl pyro-carbonate treated water (Thermo Scientific™, USA) [41]. Subsequently, cDNA synthesis was prepared using QuantiTect® Reverse Transcription kit (QIAGEN, Germany). Reaction mixture of qRT-PCR was prepared using SYBER Green (Maxima SYBR Green, Thermo Scientific™, USA), along with the primers specified in Table 1. The qRT-PCR procedure was performed using a Rotor Gene Q thermocycler (QIAGEN, Hilden, Germany) with the following program: initial denaturation at 95° C for 15 min, followed by 45 cycles of denaturation at 95 °C for 15 s, annealing for 30 s according to temperatures specified in Table 1, and extension at 72 C for 1 min. The levels of expression for the investigated genes were standardized using the 16S ribosomal RNA (rRNA) gene of *E. faecalis* as an internal standard. All measurements were performed in duplicate. The fold difference in target gene expression was calculated based on the ΔΔCt method using the cycle threshold values (Ct values). Ultimately, the relative gene expression of the experimental (polidocanol-treated) group compared to the control (untreated) group can be estimated as 2^−ΔΔCt ^ [42]. 

## Statistical analysis

Data was collected in Excel (Microsoft Office) files, and the mean and standard deviation were calculated. Data was analyzed using GraphPad Prism version 8.4.2. Statistical analysis was conducted using a one-way ANOVA, with Sidak's multiple comparisons test. All experiments were conducted three times in triplicate or quadruplicate. A significant difference between the treated and untreated groups was considered when *P* < 0.05.

## Results

### Bacterial isolation and identification

Sixty clinical isolates of *E. faecalis* were obtained from samples collected from Mansoura University Hospitals (53.4%) and Kasr Al-Ainy Hospital (46.6%). The samples were obtained from different sources, including blood (*n* = 9), pus (*n* = 6), urine (*n* = 38) and stool (*n* = 7) (Supplementary Table 2). *E. faecalis* isolates were purified on bile esculin agar medium as small black colonies with a surrounded black halo. The pure colonies were stained with Gram stain and characterized as Gram-positive small cocci in clusters or diplococci under the microscope.

Then, species identification was performed using PCR analysis through the detection of *eda1* and *eda2* genes with amplicon sizes of 404 and 389 bp, respectively which are specific to *E. faecalis* (Supplementary Fig. 1).

## Distribution of virulence factors among *E. faecalis* isolates

### Prevalence of gelatinase and protease enzymes among tested isolates

**Fig. 1:**
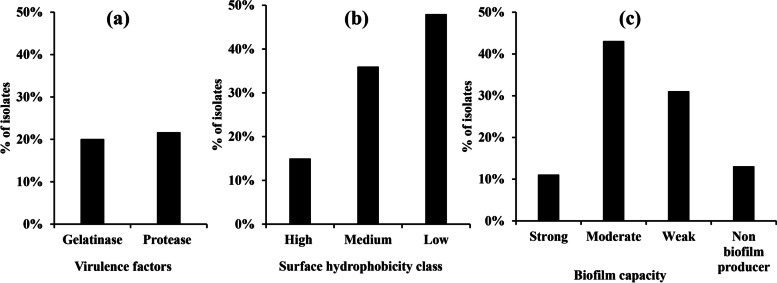
Prevalence of virulence factors among *E. faecalis* tested isolates; (**a**) Gelatinase and protease among *E. faecalis* clinical isolates. (**b**) Classification of *E. faecalis* clinical isolates according to cell surface hydrophobicity. (**c**) Capacity of biofilm formation among *E. faecalis* clinical isolates

The screening of gelatinase enzyme activity in all isolates was assessed by using gelatin agar medium. Positive gelatinase producing isolates were detected by the presence of a distinct clear halo surrounding colonies after flooding the plates with saturated ammonium sulfate solution. Among the tested isolates, 12 (20%) were found to be positive gelatinase producers (Fig. 1a and Supplementary Fig. 2a).

The proteolytic activity was detected in 13 (21.6%) of the tested isolates, as evidenced by the hydrolytic zones formed around colonies on skimmed milk agar medium resulting from the digestion of casein (Fig. 1a and Supplementary Fig. 2b).

### Detection of hydrophobicity of *E. faecalis* isolates

The cell surface hydrophobicity of *E. faecalis* isolates was assessed through evaluating the affinity towards xylene in a two-phase system. Among the tested isolates, 9 (15%) were found to be highly hydrophobic, 22 (36.6%) were moderately hydrophobic and 29 (48.3%) had a low degree of hydrophobicity (Fig. 1b).

### Biofilm production

The findings of biofilm formation by the *E.* *faecalis* isolates are displayed in Fig. 1c. The criteria for biofilm formation were elucidated as: OD ≤ 0.056 for non-adherent, 0.056 < ODi ≤ 0.112 for weakly adherent, 0.112 < ODi ≤ 0.224 for moderately adherent and ODi > 0.224 for strongly adherent. Among the *E. faecalis* isolates, 7 (11.6%) exhibited strong adherence, 27 (45%) exhibited moderate adherence, 18 (30%) showed weak adherence and only 8 (13.33%) were non biofilm producers.

## Detection of *fsr* QS and virulence genes by PCR

Four representative *E. faecalis* isolates (Ef10, Ef33, Ef35 and Ef38) were selected for their ability to produce both gelatinase and protease enzymes. Moreover, these strains exhibited biofilm formation capacity and cell surface hydrophobicity as virulence factors. PCR was used to detect *fsr* QS related genes (*fsrB* and *fsrC*), gelatinase (*gelE*), serine protease (*sprE*), as well as virulence determinant genes, pilus protein gene (*ebpA*) and aggregation substance gene (*asa1*). All QS related genes and virulence genes were amplified in the four *E. faecalis* isolates (Ef10, Ef33, Ef35 and Ef38) (Supplementary Fig. 3) and amplicons were detected at their corresponding sizes (Table 1).

## Targeting *fsr* system

A total of 66 compounds were tested to assess their ability to target *fsr* QS system in *E. faecalis* by examining their impact on gelatinase production. For preliminary screening, two *E. faecalis* isolates (Ef33 and Ef35) were chosen based on their ability for production of gelatinase enzyme. Subsequently, the potential inhibitors were also tested for their effects on other virulence factors and the expression of *fsr* QS related genes.

First, the MICs of the tested compounds were determined against *E. faecalis* isolates (Ef33 and Ef35) (Supplementary Table 1). Then, the tested compounds at sub-MICs were tested for their ability to target *fsr* QS by semi-quantitative gelatinase assay method (Supplementary Table 1). The initial screening results indicated that polidocanol could inhibit gelatinase production. The MIC of polidocanol was 4096 µg/mL against (Ef33 and Ef35). Sub-MICs (½ and ¼ MIC) of polidocanol were then used to investigate its inhibitory effect on *fsr* QS system and various virulence factors.

## Effect of polidocanol on microbial growth

**Fig. 2:**
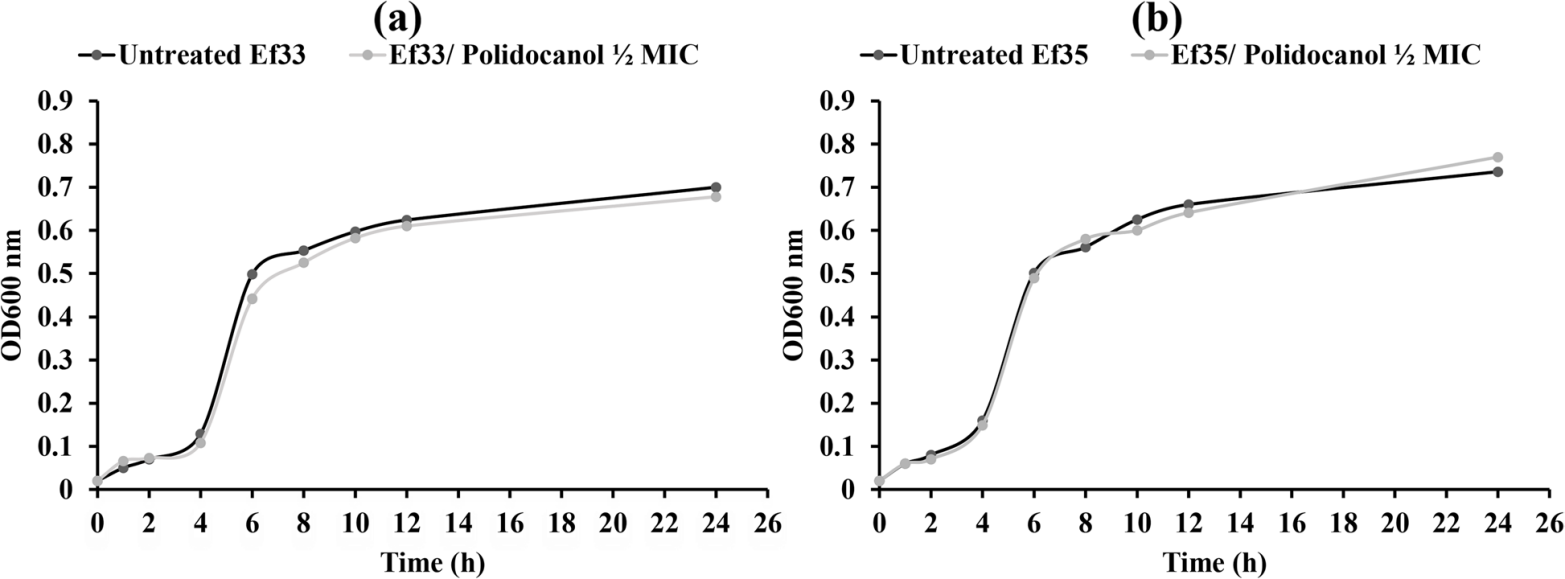
Growth curve of *E. faecalis*; (**a**) Isolate no. Ef33. (**b**) Isolate no. Ef35 in absence and presence of ½ MIC of polidocanol

The viability of *E. faecalis* was estimated after treating two isolates (Ef33 and Ef35) with ½ MIC of polidocanol. Cultivation of *E. faecalis* isolates with sub-MIC of polidocanol did not affect bacterial viability in comparison to the untreated control cultures. The count of untreated Ef33 and Ef35 was 5.6 × 10^8^ CFU/mL and 5.89 × 10^8^ CFU/mL, respectively which was not affected by treatment with ½ MIC of polidocanol, as the count of Ef33 and Ef35 treated with ½ MIC of polidocanol was 5.42 × 10^8^ CFU/mL and 6.16 × 10^8^ CFU/mL, respectively. Furthermore, the OD_600 nm_ of both treated and untreated cultures was assessed at various time intervals. The growth of bacteria in cultures treated with ½ MIC of polidocanol did not show any impact over time compared to untreated cultures (Fig. 2).

## Polidocanol inhibited *E. faecalis fsr* QS system and virulence factors

### Elimination of gelatinase production by polidocanol

The effect of polidocanol at ½ and ¼ MICs on gelatinase production from *E. faecalis* isolates Ef10, Ef33, Ef35 and Ef38 was assessed using semi-quantitative gelatin agar and quantitative azocoll methods.

**Fig. 3:**
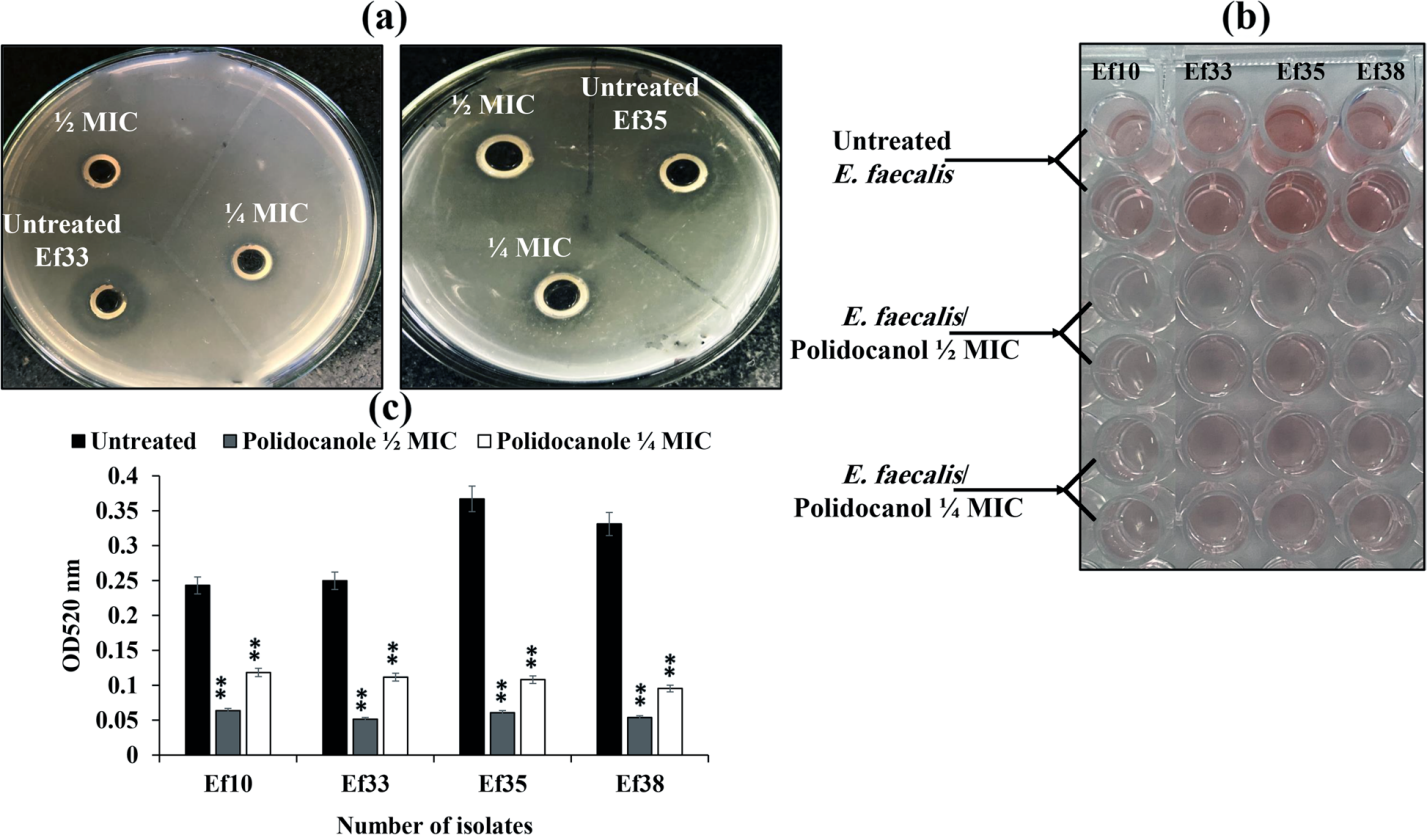
Effect of sub-inhibitory concentrations of polidocanol on gelatinase production; (**a**) Semi-quantitative assay of polidocanol impact on gelatinase production using gelatin agar plates. (**b**) Quantitative assay of the effect of polidocanol on gelatinase activity by azocoll. (**c**) Effect of sub-lethal doses of polidocanol on gelatinase production using azocoll measured at OD_520 nm_. (Each experiment was performed three times in triplicate and mean was calculated ± SD, **P < 0.01)

In the semi-quantitative gelatin agar assay, the tested isolates showed clear zones around inoculated cups indicating positive gelatinase production. However, cups filled with cell free supernatants of *E. faecalis* treated with ½ and ¼ MICs showed a loss of gelatinase activity as evidenced by the absence of clear zones (Fig. 3a).

Confirmation of these findings was obtained through the azocoll assay, which measured the degradation of azocoll in the cell free supernatant of the tested isolates. The development of a red color, measured spectrophotometrically at OD_520 nm_ indicated the presence of extracellular gelatinase enzyme. However, supernatants of isolates cultured with sub-MICs of polidocanol did not produce a red color. Treatment with ½ MIC of polidocanol resulted in significant (*P* < 0.01) reduction in gelatinase activity of isolates Ef10, Ef33, Ef35, and Ef38, ranging from 73 to 83%. Similarly, *E. faecalis* isolates treated with ¼ MIC showed a significant reduction in gelatinase activity, ranging from 51 to 71% (*P*< 0.01)** (**Figs. 3b, c).

### Inhibition of protease production by polidocanol

**Fig. 4:**
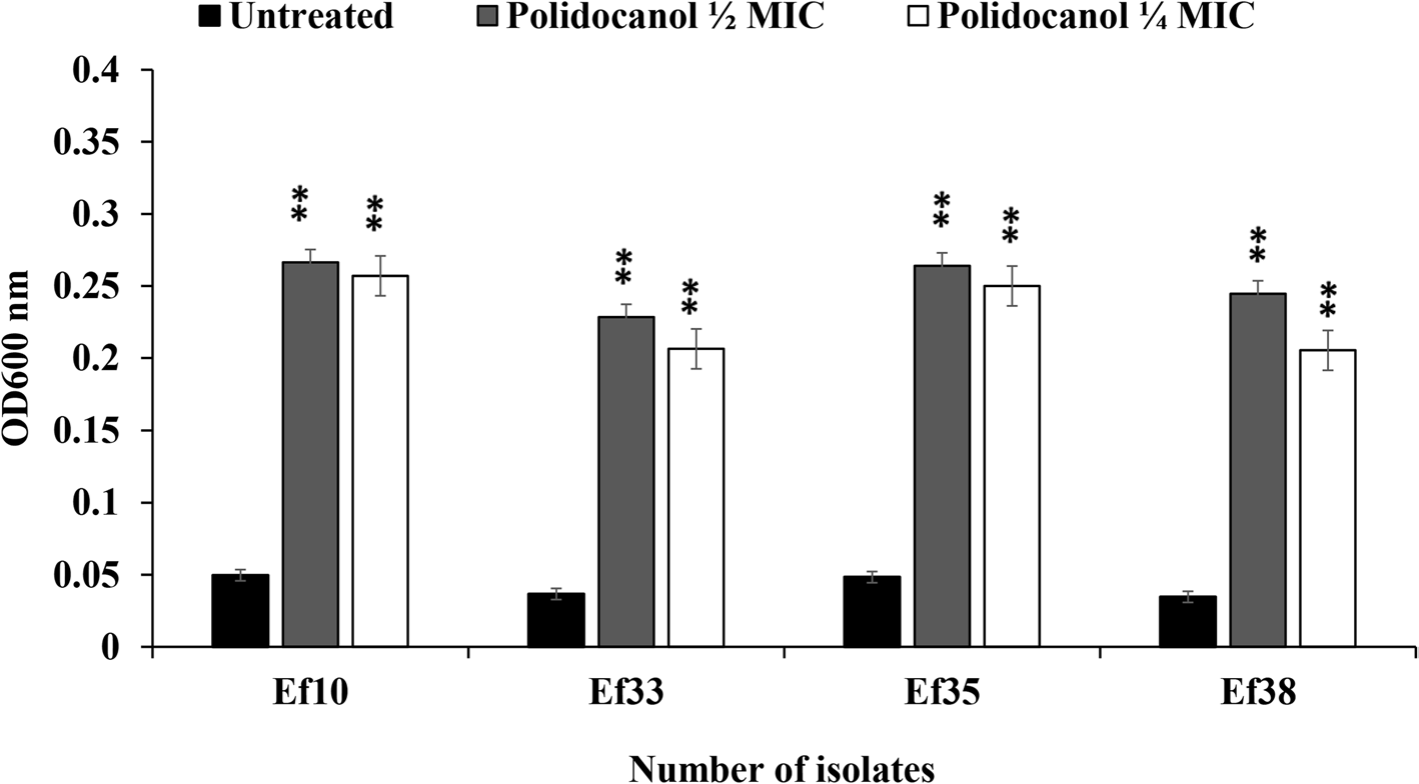
Effect of sub-MICs of polidocanol (½ and ¼ MIC) on protease production from *E. faecalis* isolates no. Ef10, Ef33, Ef35 and Ef38. (Each experiment was performed three times in triplicate and mean was calculated ± SD, **P < 0.01)

The effect of polidocanol on protease enzyme production was assessed using skimmed milk by comparing the absorbance of the supernatant from the culture grown in the presence of polidocanol to that of the control culture grown without polidocanol. Polidocanol at ½ MIC significantly decreased protease production (*P* < 0.05). The OD_600 nm_ of the supernatant of treated cultures increased by more than twofold to threefold compared to the supernatant of untreated cultures due to a decrease in the digestion of casein **(**Fig. 4**)**.

### Effect of polidocanol on cell hydrophobicity

**Fig. 5:**
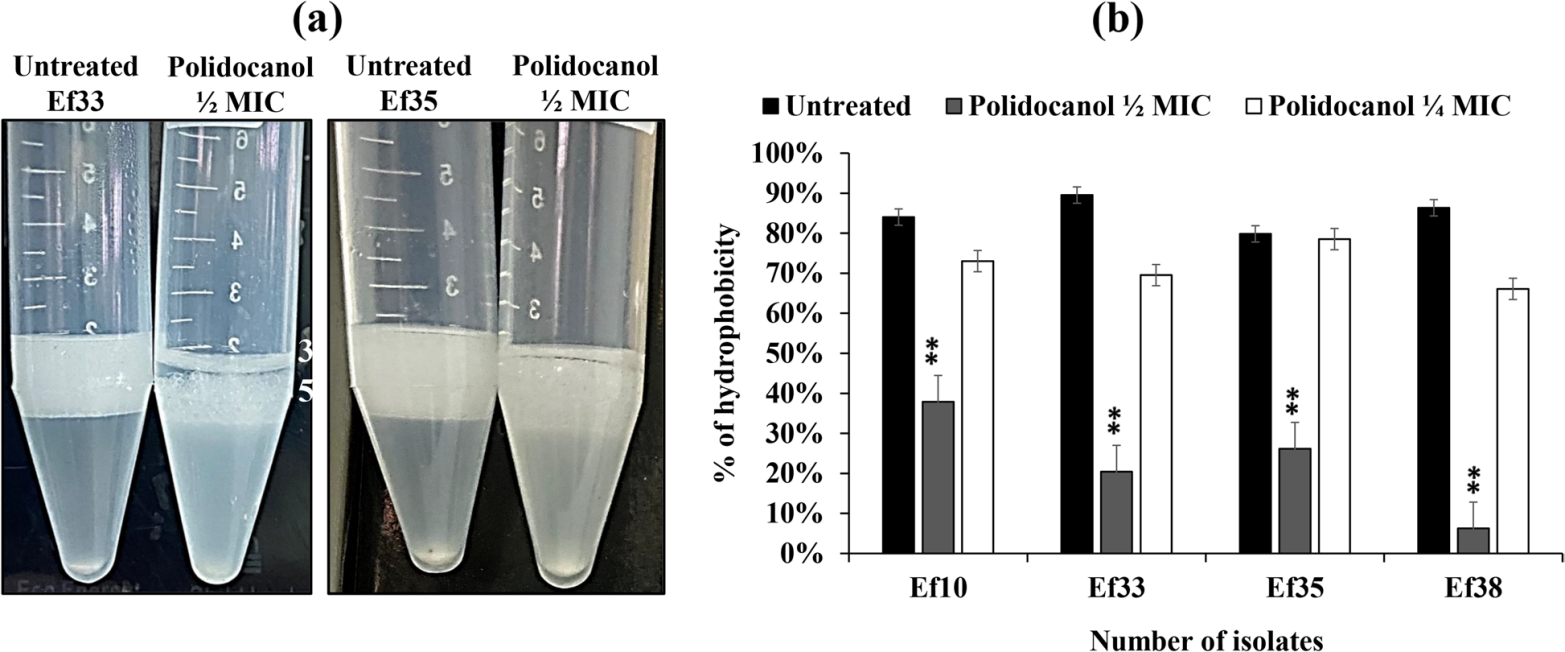
Effect of polidocanol at ½ and ¼ MIC on *E. faecalis* surface hydrophobicity; (**a**) Cell surface hydrophobicity test. (**b**) Percentage hydrophobicity of isolates treated with ¼ and ½ MIC of polidocanol compared to untreated *E. faecalis* isolates no. Ef10, Ef33, Ef35 and Ef38. (Each experiment was performed three times in triplicate and mean was calculated ± SD, **P < 0.01)

The cell surface hydrophobicity of *E. faecalis* possesses a critical function in the adhesion step during biofilm formation. To evaluate the ability of polidocanol to alter the cell surface properties, the affinity of *E. faecalis* towards xylene was tested after being exposed to sub-lethal doses of polidocanol (½ and ¼ MIC). The findings indicated that polidocanol (½ MIC) significantly decreased the hydrophobic properties of *E. faecalis* cell membrane by 46% to 80%, (*P* < 0.01) with a greater portion of bacterial cells in the aqueous phase (Fig. 5).

### Effect of polidocanol on biofilm formation

**Fig. 6:**
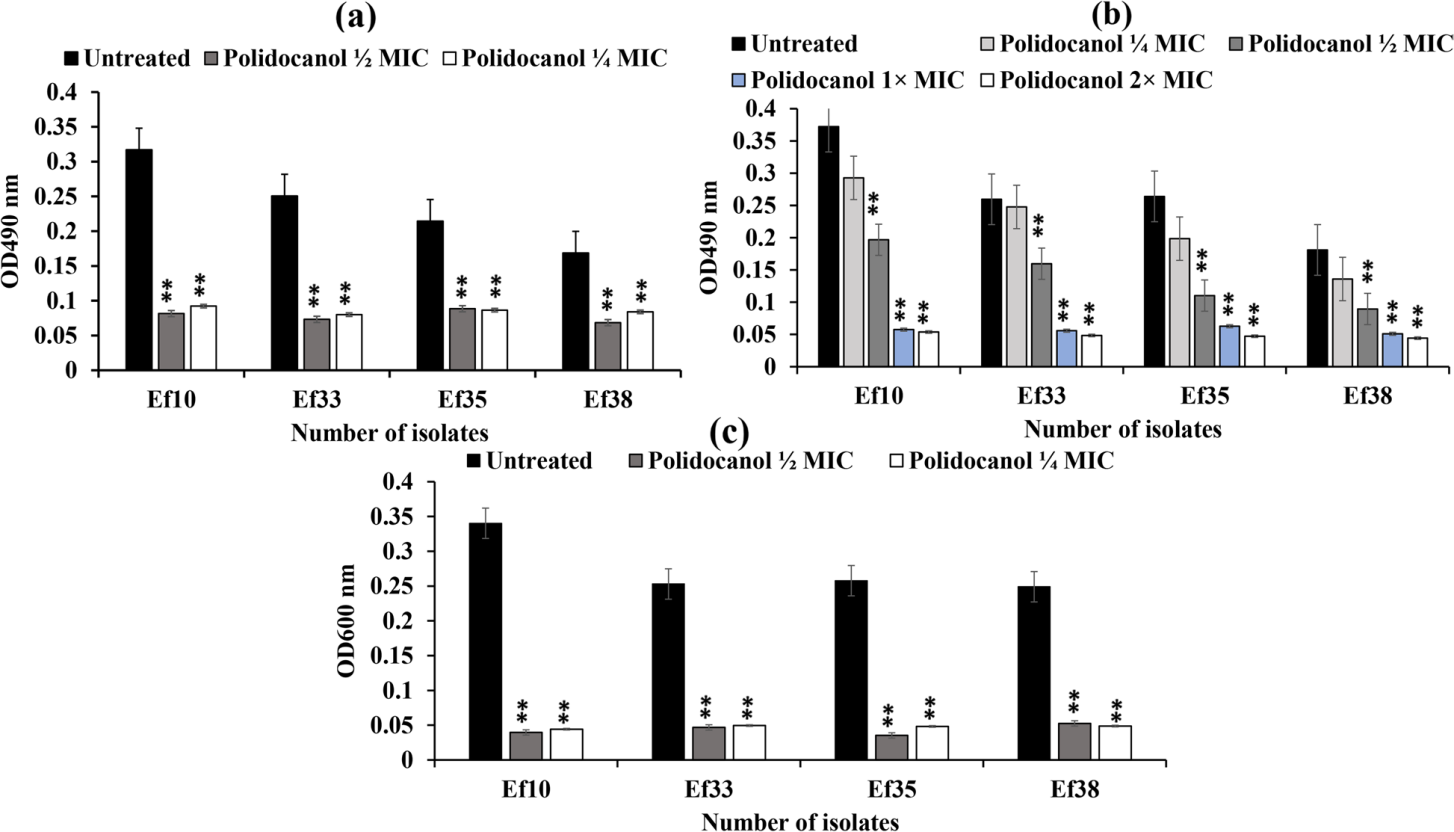
Effect of polidocanol on biofilm formation by ***E.**** faecalis*; (**a**) Biofilm inhibitory effect detected by crystal violet staining. (**b**) Biofilm eradication by polidocanol detected by crystal violet staining. (**c**) The effect of polidocanol on biofilm metabolic activity detected by tetrazolium chloride reduction assay.** (**Each experiment was performed in quadruplicate, mean was calculated ± SD, **P < 0.01)

The antagonist impact of sub-lethal concentrations of polidocanol (½ and ¼ MIC) on biofilm formation of *E. faecalis* isolates (Ef10, Ef33, Ef35 and Ef38) was tested using CV staining assay. All four isolates were biofilm producers, with isolate no. Ef10 being a strong biofilm producer, while isolates no. Ef33, Ef35 and Ef38 were moderate biofilm producers. In isolates treated with ½ and ¼ MIC of polidocanol, the capacity for biofilm formation of the tested isolates was significantly decreased by 58% to 70% (*P* < 0.01) for both concentrations (Fig. 6a).

The potential of polidocanol to eradicate mature biofilms of tested *E. faecalis* isolates (Ef10, Ef33, Ef35 and Ef38) was demonstrated using different concentrations (¼, ½, 1 × and 2 × MIC) through the CV staining method. Polidocanol at ½, 1 × and 2 × MIC significantly reduced the mature *E. faecalis* biofilms by 40% to 50%, 70% to 78% and 78% to 85%, respectively (*P* < 0.01) (Fig. 6b).

The viability of the biofilm was further estimated colorimetrically using the TTC reduction assay, which quantified the production of formazan (red color) generated by metabolically active biofilm cells. Metabolic activity within the developed biofilms by cultures of *E. faecalis* isolates (Ef10, Ef33, Ef35 and Ef38) treated with sub-MICs (½ and ¼ MIC) of polidocanol compared to control biofilms of untreated cultures was estimated. The TTC assay showed a significant decrease in metabolic activity of cells in the biofilm matrix treated with sub-MIC of polidocanol (½ and ¼ MIC) by 80% reduction with *P* value < 0.01 for both concentrations compared to untreated cells (Fig. 6c).

## Suppression of QS and virulence factors related genes

### Suppression of QS associated genes

**Fig. 7:**
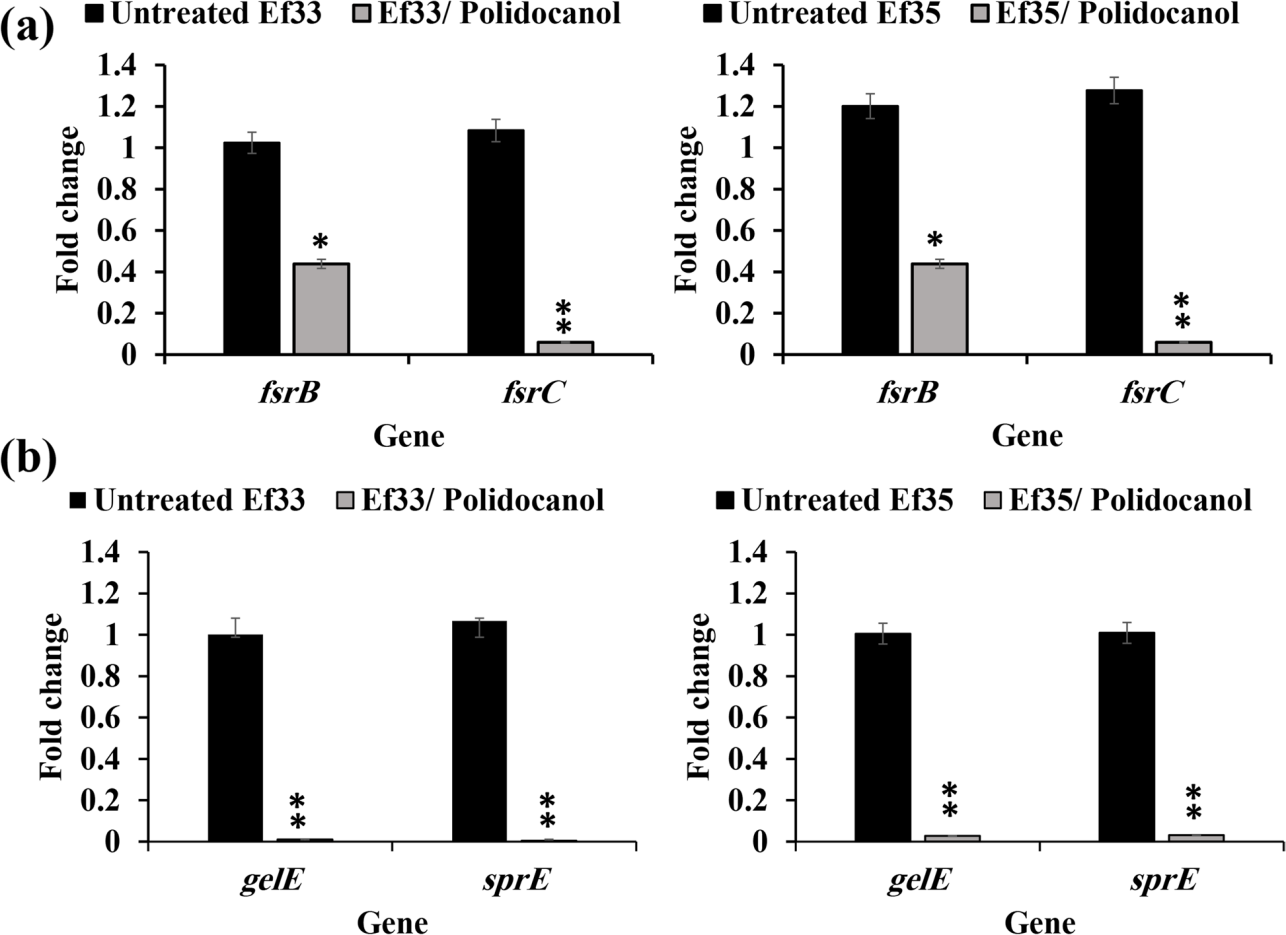
The effect of polidocanol (½ MIC) on the expression of QS related genes in *E. faecalis* isolates (Ef33 and Ef35); (**a**) QS genes (*fsrB* and *fsrC*). **b** *gel-sprE* genes, mean was calculated ± SD, *P < 0.05 and **P < 0.01)

The impact of polidocanol on the expression of genes related to the control of *fsr* QS of *E. faecalis* was investigated using qRT-PCR in isolates Ef33 and Ef35. The results showed that treatment with polidocanol (½ MIC) significantly decreased the expression of *fsr* QS system associated genes. The expression of *fsrB* was significantly reduced by 57% and 72% in isolates Ef33 and Ef35, respectively (*P* < 0.05). Additionally, more pronounced reduction in the expression of *fsrC* by 94% and 85% was observed in both isolates Ef33 and Ef35, respectively (*P* < 0.01) (Fig. 7a).

Similarly, the expression of genes encoding gelatinase (*gelE*) and protease (*sprE*) was also down regulated with *P* value < 0.01 by 97% and 96% in isolates Ef33 and Ef35, respectively **(**Fig. 7b**)**.

### Suppression of virulence associated genes

The relative expression of virulence genes that contribute to the pathogenesis of *E. faecalis* was also affected by polidocanol (½ MIC) treatment. In isolates Ef33 and Ef35, the relative expression of the pilus protein gene (*ebpA*) which contributes to the development of biofilm, decreased significantly by 92% and 97%, respectively (*P* < 0.01).

**Fig. 8:**
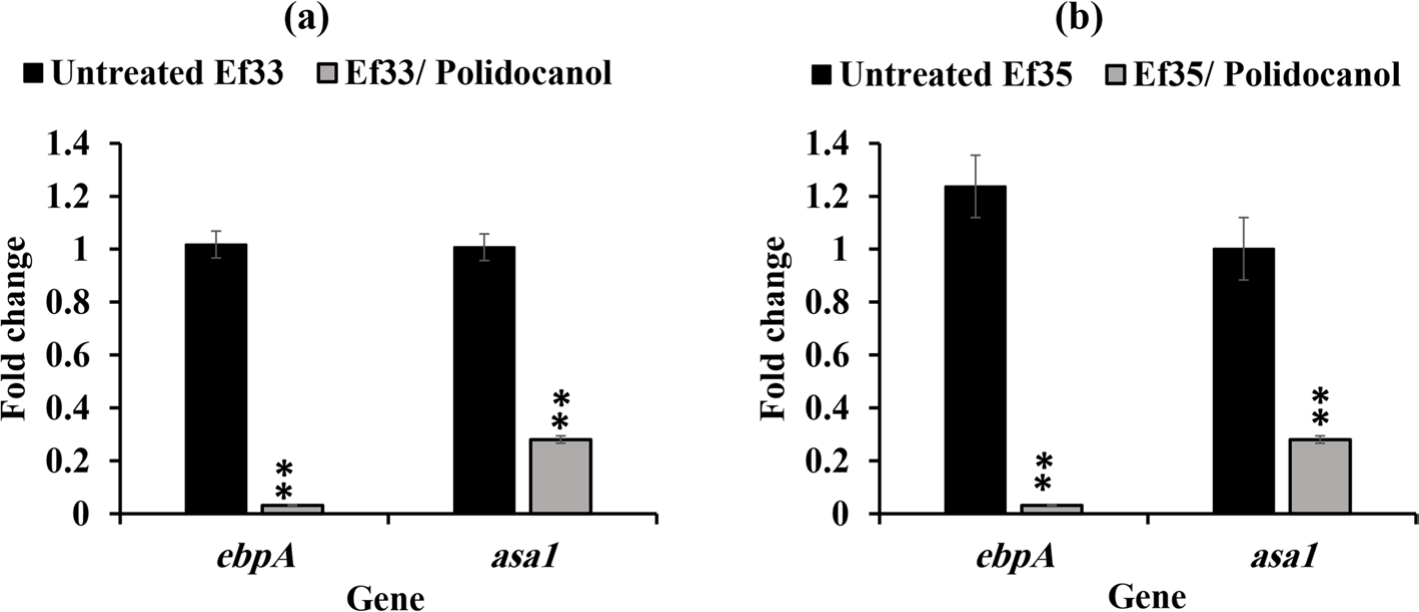
The effect of polidocanol (½ MIC) on the expression of virulence factors related genes (*ebpA* and *asa1*) in *E. faecalis* isolates; (**a**) Ef33 and (**b**) Ef35. **(**mean was calculated ± SD, **P < 0.01)

Additionally, the relative expression of the *asa1* gene which is correlated with cell aggregation was significantly reduced by 69% and 71% in isolates Ef33 and Ef35, respectively (*P* < 0.01) (Fig. 8).

## Discussion

The *fsr* QS system encoded by the *fsr* gene cluster modulates key pathogenic traits in *E. faecalis* including biofilm formation and secretion of extracellular protease and gelatinase. Therefore, with the increasing emergence of antimicrobial resistance, targeting the *E. faecalis* QS system could be an effective alternative approach to antibiotics for controlling bacterial infections. The QS inhibitors (QSIs) interfere with the bacterial ability to communicate and coordinate their virulence [43–45].

The *fsr* QS system controls the secretion of two proteases, gelatinase (GelE) and serine protease (SprE) and associated to the pathogenicity of *E. faecalis*. These proteases assist in the pathogenicity of *E. faecalis* by degrading of host tissue and facilitating the propagation of infection [46]. Additionally, GelE and SprE have been demonstrated to control bacterial autolysis and the release of extracellular DNA, thus contributing to *E. faecalis* biofilm formation [47].

In this study, 20% and 21.6% of *E. faecalis* clinical isolates produced gelatinase and protease enzymes, respectively. Similarly, gelatinase was detected in 22% of *E. faecalis* tested isolates from Egypt [8]. While, it was detected in 9.6% and 43.1% among *E. faecalis* isolates in other studies from India and Brazil, respectively [48, 49]. Gelatinase contributes to virulence by degrading of collagen, fibrinogen, fibrin and certain complement components C3 and C3a which facilitate tissue invasion, impair host defenses and promote the persistence of infection [47].

For additional characterization of *E. faecalis*, the cell surface hydrophobicity of the *E. faecalis* isolates is essential for bacterial adherence and biofilm formation. It was analyzed by the microbial adhesion to hydrocarbon layer. Examination of the hydrophobic characteristics of the cell surface of *E. faecalis* showed that 15%, 36% and 48% of the tested isolates were high hydrophobic, moderate hydrophobic and hydrophilic, respectively. At the same instance, high and moderate hydrophobicity was detected in 33% of *E. faecalis* isolated from free-living birds [36].

Biofilm formation is a significant virulence characteristic of *E. faecalis* that plays a role in the initiation and dissemination of enterococcal infections. Biofilm production confers resistance to antibiotics and the host immune response [50]. The majority of the *E. faecalis* clinical isolates had a moderate ability for biofilm formation (45%) followed by 30% of isolates that were weak biofilm producers and 11.6% that were strong producers. These results are comparable to those of Lopez and coauthors who concluded that most *E. faecalis* tested isolates purified from different clinical specimens were moderate biofilm producers (48%), while 36% were low biofilm producers, and 4.6% of tested isolates were strong biofilm producers [51].

In this study, it was observed that around half of the protease-producing *E. faecalis* isolates exhibited moderate biofilm formation, with 8% showing strong biofilm production. Hashem and colleagues also noted that 40% of gelatinase-producing *E. faecalis* clinical isolates displayed moderate biofilm formation, while 26% were strong biofilm producers [52]. In terms of cell surface hydrophobicity, 41.6% of protease producers exhibited moderate hydrophobic characteristics, while 25% displayed strong hydrophobic traits. Previous studies have suggested a significant role of gelatinase activity in enhancing bacterial cell surface hydrophobicity in *E. faecalis* [53, 54]. Furthermore, *E. faecalis* isolates that were moderate and strong biofilm producers also showed hydrophobic cell surface properties, consistent with the findings of a study by Stępień-Pyśniak and colleagues, which linked biofilm formation in *E. faecalis* isolates from birds to surface hydrophobicity [36].

In an attempt to control the pathogenesis of *E. faecalis* by interfering with the *fsr* QS that regulates significant virulence factors, a random screening approach was used to assess the WHO approved compounds and identify novel QSI activity [55]. A total of 66 compounds were screened for targeting the *fsr* QS system. As gelatinase production is one of the main virulence factors that can be altered by QS [56], preliminary screening was performed by evaluating the effect of tested compounds on gelatinase activity using a semi-quantitative method [31]. The MIC of each compound was detected by the micro broth dilution method. Subsequently, sub-MICs of each compound were used for the initial screening of gelatinase activity.

In the preliminary screening, polidocanol, was found to decrease gelatinase activity. Polidocanol is an FDA-approved sclerosant indicated for treating uncomplicated spider veins and reticular veins in the lower extremities [57].

This result was confirmed by a second quantitative assessment of gelatinase production using azocoll. Polidocanol significantly decreased gelatinase activity at ½ and ¼ MIC (*P* < 0.01) without affecting bacterial growth. The culture treated with ½ MIC of polidocanol had the same growth curve as the untreated culture of *E. faecalis* as both treated and untreated cells reached the exponential growth at the same time. In a study by Desouky and colleagues, 7 out of 54 compounds of actinomycetes metabolites decreased gelatinase activity by 80% through targeting *fsr* QS in *E. faecalis* [44].

Furthermore, polidocanol at ½ and ¼ MIC significantly reduced protease activity. The inhibitory effect of polidocanol on protease activity could be attributed to targeting *fsr* QS. Previous studies have shown that interfering with *fsr* QS was associated with reduction in *E. faecalis* protease production [33, 43]. Cinnamaldehyde at ½ MIC reduced protease activity due to its effect on *fsr* QS [58]. Additionally, Salvadora persica extract showed QSI activity against *E. faecalis* with a reduction in protease activity [43].

Furthermore, Polidocanol (½ MIC) showed a significant decrease (*P* < 0.01) in hydrophobicity characteristics. This observed decrease could be attributed to the capability of polidocanol to alter the cell surface properties and to decrease the expression of one of the surface proteins [59]. Several studies have investigated that the hydrophobicity of the enterococcal cell surface is increased by the expression of aggregation substances regulated by aggregation genes such *asa1* [31, 36]. Furthermore, the gelatinase enzyme can increase cell surface hydrophobicity by cleaving surface polypeptides at hydrophobic residues. Therefore, the attenuation of the *fsr* QS system may result in a reduction in surface hydrophobicity [60].

Additionally, Polidocanol at sub-MICs significantly decreased biofilm formation and eliminated mature biofilms at ¼, ½, 1 × and 2 × MICs (*P* < 0.01) in a dose dependent response. The metabolic activity of biofilms significantly reduced (*P* < 0.01) at sub-MICs of polidocanol**.** This could be attributed to the inhibition of *gelE* and *fsr* regulated biofilm formation as the knockout of *gelE* or *fsr* resulted in a decrease in biofilm formation [54]. The study of Suttipalin and colleagues indicated that the inhibition and eradication of *E. faecalis* biofilms by curcuminoids were related to the inhibition of gelatinase activity [61]. Similarly, Akshaya and coauthors reported that inhibition of biofilm formation and viability by cinnamaldehyde was due to the inhibition of gelatinase production [58]. According to previous findings, the biofilm inhibitory potential of polidocanol may be linked to a reduction in gelatinase activity, as well as a decrease in cell surface hydrophobicity. This conclusion is supported by a study conducted by Fu et al., which indicated that diacerein can also inhibit biofilm formation by reducing cell surface hydrophobicity [62].

Based on the results of the observed phenotypes, the effect of polidocanol on the expression of QS and virulence genes was investigated using qRT-PCR. In the QS circuit of *E. faecalis*, the *fsrB* gene encodes a transmembrane protein that processes a propeptide to produce a peptide pheromone. The *fsrC* gene encodes a histidine kinase sensor that responds to the peptide-signaling molecule, phosphorylates its response regulator, and then activates the *gelE-sprE* encoding gelatinase and serine protease enzymes [7]. Polidocanol at ½ MIC significantly down regulated *fsrB* (*P* < 0.05) and *fsrC* (*P* < 0.01) gene expression in *E. faecalis* isolates Ef33 and Ef35. In turn, the expression of *gelE-sprE* was significantly reduced (*P* < 0.01) in Ef33 and Ef35 which could be attributed to the inhibitory effect of polidocanol on *fsrB* and *fsrC*. Deletion of *fsrA, fsrB*, or *fsrC* in the wild-type *E. faecalis* strain OG1RF inhibits the expression of *gelE* and *sprE* [63]*.* This supports the potential QSI activity of polidocanol and its ability to disrupt the QS pathway in *E. faecalis*. The findings from the gene expression analysis were consistent with the results of gelatinase activity. Similarly, Islam and coauthors found that trans-cinnamaldehyde at sub-MIC concentration attenuated the *fsr* QS system by downregulation of *fsrB* and *fsrC* QS genes led to downregulation of *gelE* gene [33].

Suppression of the *fsr* QS system and inhibition of the gelatinase production have been proposed as critical factors that reduce the destruction of host tissue components caused by bacterial colonization. Many studies have proved that biofilm formation in *E. faecalis* can be managed by QS [64]. Downregulation of QS related genes (*gelE*-*sprE*) leads to disruption and inhibition of biofilm formation [65].

Besides *gelE* and *sprE* regulation, it has been revealed that QS systems also indirectly control other virulence genes included in surface adhesion and aggregation substances [7]. The activity of polidocanol against the *fsr* QS system could explain its inhibitory effect on gene expression of other *E. faecalis* virulence factors. The qRT-PCR results revealed that polidocanol significantly decreased the expression of the virulence genes *ebpA* and *asa1* (*P* < 0.01). Aggregation substance is a surface protein encoded by the *asa1* gene playing a crucial function in biofilm formation and adherence to host tissues, an important factors in pathogenesis [20]. The EbpA protein is a cell wall-anchored protein encoded by the *ebpA* gene. EbpA is a subunit of the endocarditis and biofilm associated pilus (Ebp), which is involved in the formation of biofilm and the development of endocarditis [66]. Attenuating the expression of *ebpA* resulted in decreased biofilm formation and adherence to fibrinogen in vitro, suggesting that EbpA is important for the formation and stability of the Ebp pilus and bacterial attachment to host tissues [67]. Therefore, polidocanol has the potential to reduce *E. faecalis* pathogenicity by targeting the *ebpA* and *asa1* genes.

There are many hypotheses for inhibition of *fsr* QS pathway including blocking the production of GBAP, non-competitive inhibition of GBAP or disturb signal transduction of the two-component regulatory system and that may be related to structural activity. Further investigations are needed to elucidate the potential mechanism of QS inhibition of polidocanol.

## Conclusion

The findings of this study display the ability of polidocanol to interfere with *fsr* quorum sensing system in *E. faecalis* without affecting bacterial growth*.* This inhibitory effect could be attributed to the sub- inhibitory concentration of polidocanol on the relative expression of QS genes. The reduction in the relative expression of *fsrB* and *fsrC* QS genes was associated with a significant suppression in *gelE-sprE* expression. Interference with the QS system was associated with a significant inhibition of virulence traits including gelatinase activity, protease activity, cell surface hydrophobicity and biofilm formation. Furthermore, it decreased the relative expression of *ebpA* and *asa1* virulence genes. Overall, these findings suggest that polidocanol can reduce the pathogenesis and dissemination of *E. faecalis* infection via QS interference. Additional research is needed to explore its in vivo antipathogenic effect.

## Supplementary Information

Supplementary Material 1.

## Data Availability

All data developed or analysed during this research are provided in the manuscript, the supplementary information files and available from the corresponding author on reasonable request.

## Abbreviations

*E. **faecalis*: *Enterococcus* *faecalis*
*Fsr*: *Enterococcus**faecalis* System regulator
QS: Quorum sensing
GBAP: Gelatinase biosynthesis-activating pheromone
GelE: Gelatinase
SprE: Serine proteas
Sub-MIC: Subinhibitory concentration
OD: Optical density
PBS: Phosphate buffer
TTC: Tetrazolium chloride
TSB: Tryptic soy broth
qRT-PCR: Quantitative Real-time Polymerase Chain reaction
Ct: Cycle threshold

## Data availability

No datasets were generated or analysed during the current study.
