# Supplementary material for: Polidocanol inhibits Enterococcus faecalis virulence factors by targeting fsr quorum sensing system
Source: BMC Microbiol. 2024 Oct 16;24:411. doi: 10.1186/s12866-024-03548-2 (PMC11481293; doi:10.1186/s12866-024-03548-2)
Supplement: Supplementary file 1 — Supplementary Material 1. [file 12866_2024_3548_MOESM1_ESM.pdf]

Polidocanol Inhibits *Enterococcus faecalis* Virulence Factors by Targeting *Fsr* Quorum Sensing System

Dina Ashraf, Abeer M. Abd El-Aziz, Mona I. Shaaban*, Ramadan Hassan

Department of Microbiology and Immunology, Faculty of Pharmacy, Mansoura University, Mansoura 35516, Egypt

***Corresponding author:** Mona I. Shaaban

mona_ibrahem@mans.edu.eg

(a)

(b)

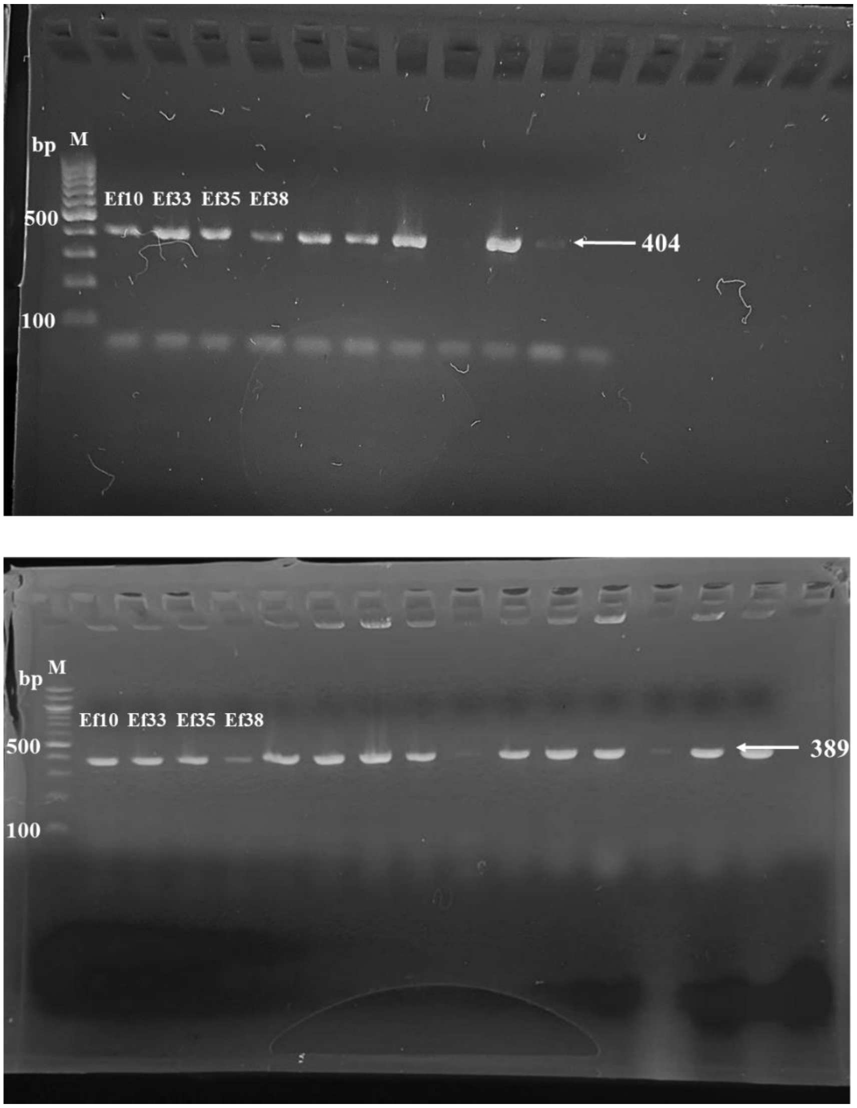

Supplementary fig 1. Amplification of species specific genes of *E. faecalis*; (a) *eda1* gene (404 bp) **(b)** *eda2* gene (389 bp). Lane M is 100 bp DNA marker.

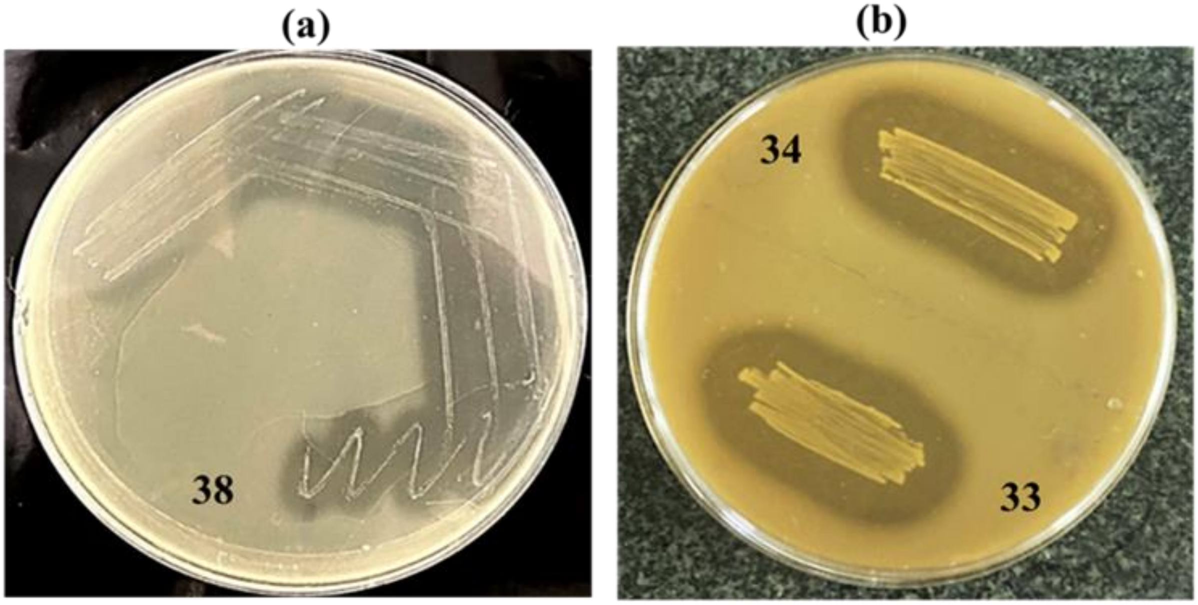

Supplementary fig 2. Phenotypic detection of; (a) Gelatinase enzyme on gelatin agar medium. **(b)** Protease enzyme on skimmed milk agar medium.

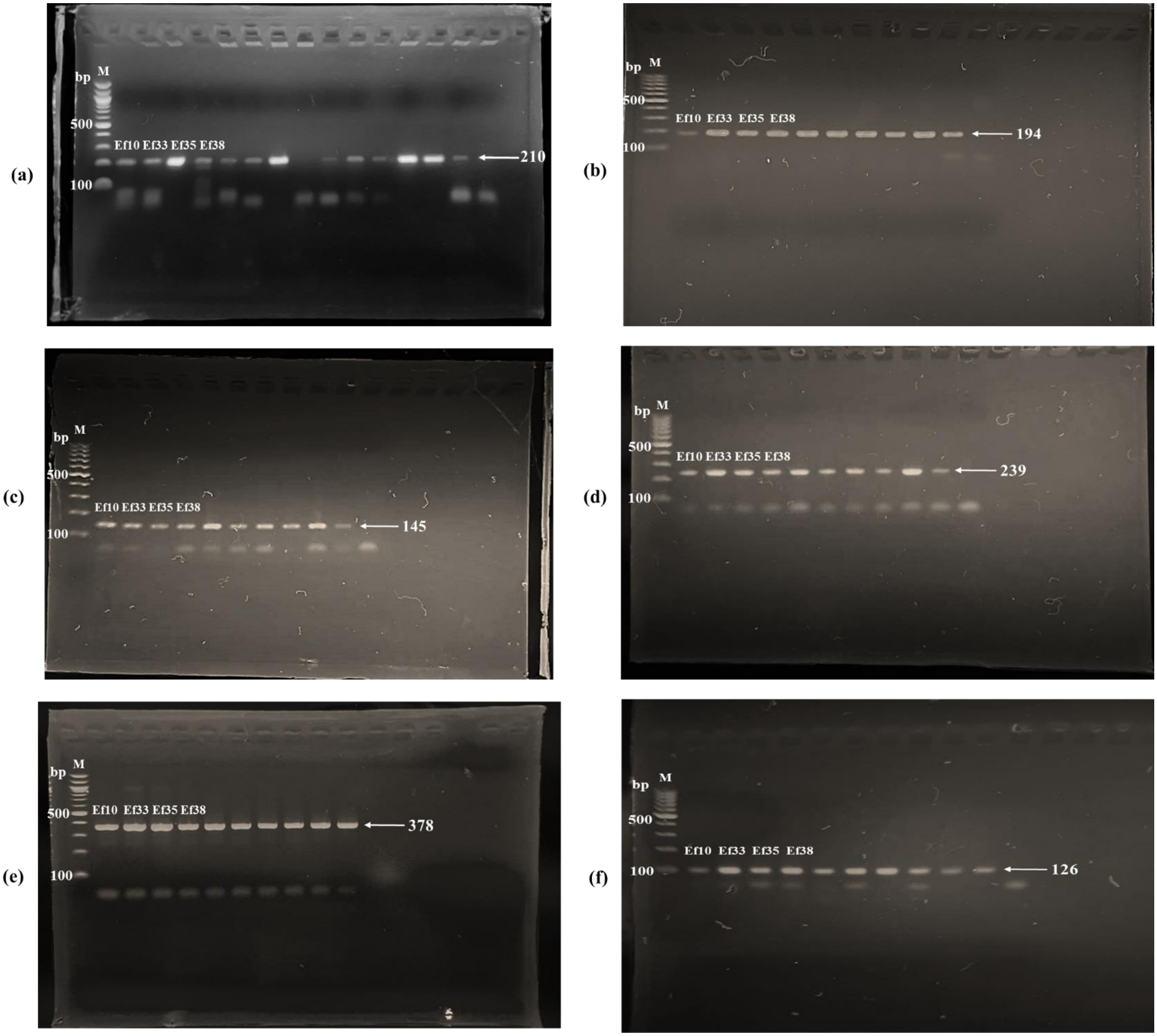

Supplementary fig 3. Amplification of *fsr* QS genes and virulence genes in *E. faecalis* isolates by PCR; (a) *fsrB* gene (210 bp) **(b)** *fsrC* gene (194 bp) **(c)** *gelE* gene (145 bp) **(d)** *sprE* gene (239 bp) **(e)** *asa1* gene (378 bp) **(f)** *ebpA* gene (126 bp). Lane M is 100 bp DNA marker.

Supplementary table 1. Compounds used for screening of *fsr* QS inhibition

Compound name	Average MIC (µg/mL)	Semi quantitative assay of gelatinase	Compound name	Average MIC (µg/mL)	Semi quantitative assay of gelatinase
**1. Caffeine**	8192	**-**	**34. Atracurium besylate**	4096	**-**
**2. Codeine**	4096	**-**	**35. Midazolam**	4096	**-**
**3. Colistin**	2048	**-**	**36. Salbutamol**	256	**-**
**4. Cansidas**	512	**-**	**37. Propranolol HCl**	512	**-**
**5. Amphotericin B**	4096	**-**	**38. Ephedrine**	8192	**-**
**6. Atropine**	2048	**-**	**39. Tiemonium**	2048	**-**
**7. Hyoscyamine**	1024	**-**	**40. Dobutamine**	8192	**-**
**8. Heparin**	8192	**-**	**41. Adrenaline**	256	**-**
**9. Phytonadione**	8192	**-**	**42. Diazepam**	2048	**-**
**10. Doxycycline**	16384	**-**	**43. Haloperidol**	256	**-**
**11. Hydroxocobalamin**	512	**-**	**44. Hyoscine**	16384	**-**
**12. Amikacin**	128	**-**	**45. Diclofenac**	16384	**-**
**13. Gentamycin**	64	**-**	**46. Piroxicam**	1024	**-**
**14. Ciprofloxacin**	32768	**-**	**47. Meloxicam**	2048	**-**
**15. Cefoxitin**	16384	**-**	**48. Ketorolac**	4096	**-**
**16. Ceftriaxone**	8192	**-**	**49. Triamcinolone acetonide**	8192	**-**
**17. Ceftazidime**	16384	**-**	**50. Dexamethasone**	4096	**-**
**18. Clindamycin**	65536	**-**	**51. Bumetanide**	128	**-**
**19. Metronidazole**	4096	**-**	**52. Furosemide**	8192	**-**
**20. Troxerutin**	16384	**-**	**53. Torsemide**	4096	**-**
**21. Phentolamine**	2048	**-**	**54. Tranexamic acid**	65536	**-**
**22. Pheniramine**	8192	**-**	**55. Pentoxifylline**	8192	**-**
**23. Cyclizine lactate**	512	**-**	**56. Carbetocin**	65536	**-**
**24. Famotidine**	8192	**-**	**57. Sulfonic acid**	32768	**-**
**25. Ethanolamine**	16384	**-**	**58. L-carnitine**	131072	**-**
**26. Etoposide**	8192	**-**	**59. Neostigmine**	256	**-**
**27. Methotrexate**	16384	**-**	**60. Acefylline piperazine**	65536	**-**
**28. Granisetron**	512	**-**	**61. Trimebutine maleate**	2048	**-**
**29. Levetiracetam**	16384	**-**	**62. Digoxin**	65536	**-**
**30. Phenytoin**	8192	**-**	**63. Calcitonin**	4096	-
**31. Cerebrolysin**	131072	**-**	**64. Polidocanol**	2048	+
**32. Piracetam**	131072	**-**	**65. Drotaverine**	16384	**-**
**33. Meclofenoxate**	2048	**-**	**66. Metoclopramide**	4096	**-**

Supplementary table 2. Clinical sources of the tested *E. faecalis* isolates

Isolate code	Clinical source	Isolate Code	Clinical source
**1**	Urine	**31**	Stool
**2**	Urine	**32**	Stool
**3**	Urine	**33**	Stool
**4**	Pus	**34**	Stool
**5**	Urine	**35**	Stool
**6**	Urine	**36**	Urine
**7**	Urine	**37**	Urine
**8**	Urine	**38**	Urine
**9**	Urine	**39**	Urine
**10**	Blood	**40**	Urine
**11**	Urine	**41**	Urine
**12**	Pus	**42**	Urine
**13**	Pus	**43**	Urine
**14**	Blood	**44**	Urine
**15**	Urine	**45**	Urine
**16**	Urine	**46**	Urine
**17**	Urine	**47**	Urine
**18**	Urine	**48**	Urine
**19**	Urine	**49**	Urine
**20**	Pus	**50**	Urine
**21**	Blood	**51**	Urine
**22**	Urine	**52**	Urine
**23**	Urine	**53**	Urine
**24**	Urine	**54**	Blood
**25**	Urine	**55**	Blood
**26**	Pus	**56**	Blood
**27**	Urine	**57**	Blood
**28**	Urine	**58**	Blood
**29**	Stool	**59**	Blood
**30**	Stool	**60**	Pus
